# Supplementary material for: Comparative Analysis of Bisexual and Parthenogenetic Populations in Haemaphysalis Longicornis
Source: Microorganisms. 2024 Apr 19;12(4):823. doi: 10.3390/microorganisms12040823 (PMC11051975; doi:10.3390/microorganisms12040823)
Supplement: Supplementary file 1 [file microorganisms-12-00823-s001.zip › microorganisms-2945637-supplementary.pdf]

Supplementary Materials:

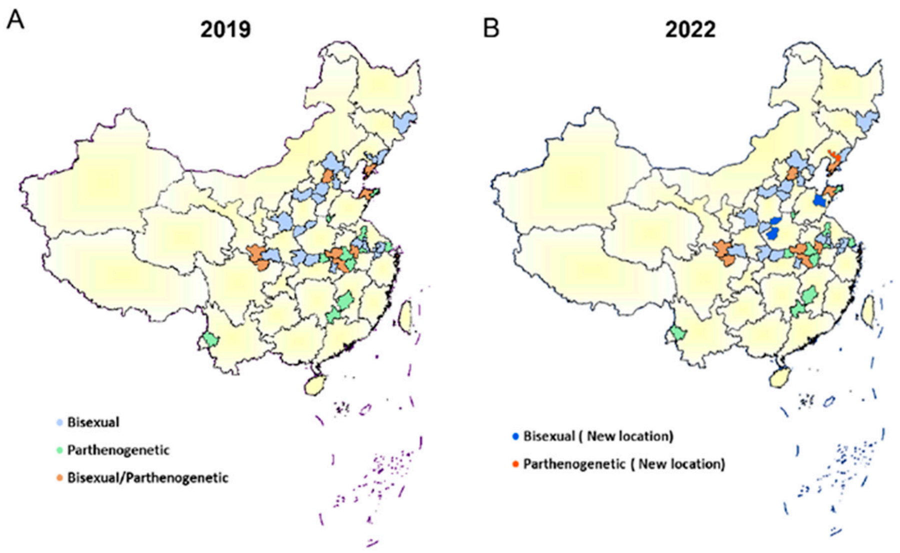

Figure S1. Geographical distribution of bisexual and parthenogenetic *Haemaphysalis longicornis* collected in China from 2019 to 2022. (**A**) Distribution of bisexual and parthenogenetic *H. longicornis* in China in 2019. Green areas indicate parthenogenetic *H. longicornis*, blue areas indicate bisexual *H. longicornis*, brown areas indicate both bisexual and parthenogenetic *H. longicornis*. (**B**) Distribution of bisexual and parthenogenetic *H. longicornis* in China in 2022. Orange areas indicate newly discovered parthenogenetic *H. longicornis*, dark blue areas indicate newly discovered bisexual *H. longicornis* relative to 2019.
